# Quinolone resistance in *Riemerella anatipestifer* from Thai ducks: Mutation analysis of *gyrA, parC*, and *plasmid-mediated quinolone resistance* genes

**DOI:** 10.14202/vetworld.2025.1891-1898

**Published:** 2025-07-11

**Authors:** Chutima Pathomchai-Umporn, Sudtisa Laopiem, Kriangkrai Witoonsatian, Sittinee Kulprasetsri, Pun Panomwan, Thaweesak Songserm, Nuananong Sinwat

**Affiliations:** 1 Department of Farm Resources and Production Medicine, Faculty of Veterinary Medicine, Kasetsart University, Kamphaeng Saen Campus, Nakhon Pathom, 73140 Thailand; 2 Department of Pathology, Faculty of Veterinary Medicine, Kasetsart University, Kamphaeng Saen Campus, Nakhon Pathom, 73140 Thailand

**Keywords:** ducks, plasmid-mediated quinolone resistance, quinolone resistance-determining region, *Riemerella anatipestifer*

## Abstract

**Background and Aim:**

*Riemerella anatipestifer* is a Gram-negative bacterium causing systemic infections in ducks, often treated with quinolones. However, increasing resistance to quinolones poses a threat to effective treatment, and the molecular mechanisms underlying this resistance remain inadequately understood in Thailand. This study aimed to determine the minimum inhibitory concentrations (MICs) of nalidixic acid, ciprofloxacin, and enrofloxacin; identify mutations in the quinolone resistance-determining regions of *gyrA* and *parC*; and detect *plasmid-mediated quinolone resistance* (*PMQR*) genes in *R. anatipestifer* isolates from Thai ducks.

**Materials and Methods:**

A total of 37 clinical isolates of *R. anatipestifer* were collected from diseased ducks between 2021 and 2023. MICs were determined using the agar dilution method, following the guidelines of the Clinical and Laboratory Standards Institute. Polymerase chain reaction and Sanger sequencing were employed to detect mutations in *gyrA* and *parC* and to screen for *PMQR* genes (*qnrA*, *qnrB*, and *qnrS*). Phylogenetic analysis of the *gyrA* gene was performed to assess the relatedness among isolates.

**Results:**

Nalidixic acid MICs ranged from 16 μg/mL to ≥128 μg/mL; ciprofloxacin from 1 μg/mL to 8 μg/mL; and enrofloxacin from 0.25 μg/mL to 4 μg/mL. All isolates had a single point mutation at codon 83 of *gyrA*, either C248T (Ser83Ile, n = 35) or C248G (Ser83Arg, n = 2). No mutations were observed in *parC*, and none of the *PMQR* genes were detected. Phylogenetic analysis grouped most Thai isolates into one major cluster, with a few aligning with Chinese strains and the American Type Culture Collection reference strain.

**Conclusion:**

This study provides the first molecular evidence of quinolone resistance mechanisms in *R. anatipestifer* from ducks in Thailand. Resistance appears primarily associated with a single mutation at codon 83 of *gyrA*, while no *parC* mutations or *PMQR* genes were detected. These findings highlight the importance of ongoing resistance surveillance and prudent antimicrobial use. Despite limitations in sample size and gene scope, this study provides essential baseline data to inform treatment guidelines and supports the inclusion of *R. anatipestifer* monitoring in Thailand’s national antimicrobial resistance action plan. Future research should explore additional resistance genes using advanced genomic tools.

## INTRODUCTION

*Riemerella anatipestifer* is a Gram-negative bacterium within the family *Flavobacteriaceae* [1]. Although not classified as zoonotic, it has a significant impact on poultry health, especially in ducks [2]. Infected ducks often exhibit respiratory distress and neurological symptoms, resulting in reduced growth rates, decreased egg production, and increased mortality, particularly in cases of co-infection with other pathogens. Due to the limited efficacy of vaccines across different *R. anatipestifer* serotypes, antibiotic therapy remains a cornerstone of disease control in poultry farms [3].

Among antibiotics, quinolones have been widely used over the past decade for treating *R. anatipestifer* infections. However, their extensive and often indiscriminate use has likely contributed to the emergence of quinolone resistance in this pathogen. The increasing prevalence of quinolone-resistant Gram-negative bacteria in poultry presents a growing threat to animal health management worldwide [4]. Despite this concern, limited information is available regarding the specific resistance mechanisms in *R. anatipestifer*.

Quinolone resistance typically involves multiple mechanisms, including mutations in quinolone resistance-determining regions (QRDRs), efflux pump overexpression, and the acquisition of *plasmid-mediated quinolone resistance* (*PMQR*) genes. Mutations within the QRDRs of *gyrA* and *parC*, which encode the subunits of DNA gyrase and topoisomerase IV, respectively – enzymes critical for bacterial DNA replication and transcription – are the most common resistance-conferring alterations [5]. Such amino acid substitutions, particularly in GyrA and ParC, are frequently associated with high-level fluoroquinolone resistance in various Gram-negative pathogens [6, 7].

Beyond chromosomal mutations, *PMQR* genes also play a significant role in the dissemination of resistance. Their presence in both clinical and environmental isolates underscores public health concerns due to their potential for horizontal gene transfer. These genes enable the rapid spread of resistance among Gram-negative bacteria and may compromise the efficacy of quinolones in both veterinary and human medicine [8].

Despite the rising global concern over anti- microbial resistance in Gram-negative bacteria, particularly in poultry pathogens, limited research has been conducted on the molecular mechanisms underlying quinolone resistance in *R. anatipestifer*, especially in Thailand. Most existing studies have focused on phenotypic resistance patterns or have been geographically confined to China and select regions. Furthermore, while mutations in the QRDRs of *gyrA* and *parC* and the presence of PMQR genes have been implicated in resistance, their prevalence and molecular characterization in Thai *R. anatipestifer* isolates remain poorly understood. The lack of region-specific molecular data impedes the development of effective diagnostic tools, surveillance strategies, and antimicrobial stewardship policies tailored to the Thai duck production system.

This study aimed to characterize the quinolone resistance mechanisms in *R. anatipestifer* isolates obtained from diseased ducks in Thailand. Specifically, it sought to (i) determine the minimum inhibitory concentrations (MICs) of nalidixic acid, ciprofloxacin, and enrofloxacin; (ii) identify mutations within the QRDRs of the *gyrA* and *parC* genes; and (iii) screen for the presence of PMQR genes (*qnrA*, *qnrB*, and *qnrS*). By integrating phenotypic susceptibility testing with molecular and phylogenetic analyses, the study provides foundational insights into the genetic basis of quinolone resistance in *R. anatipestifer* and supports evidence-based interventions for antimicrobial resistance (AMR) management in the Thai poultry sector.

## MATERIALS AND METHODS

### Ethical approval

All procedures for sample collection from diseased ducks in this study were approved by the Kasetsart University Institutional Animal Care and Use Committee (ACKU66-VET-072) and found to be in accordance with the guidelines of animal care and use established by the Ethical Review Board of the Office of National Research Council of Thailand for the conduct of scientific research. The committee approved and permitted animal care and use as outlined in the study and animal use protocol.

### Study period and location

The study was conducted from January 2021 to December 2023. Diseased ducks from Thailand’s central region were submitted to the Kasetsart Veterinary Diagnostic Center, Faculty of Veterinary Medicine, Kasetsart University, Kamphaeng Saen Campus, Nakhon Pathom for necropsy and laboratory diagnosis.

### Sample collection and bacterial isolation

Thirty-seven *R. anatipestifer* isolates originated from clinical cases in ducks exhibiting respiratory distress or neurological symptoms, with necropsy findings revealing fibrinopurulent polyserositis lesions in visceral organs, including the liver, heart, and brain. The tissue samples were aseptically collected and immediately sent to the microbiology laboratory for bacterial culture within 1 h of sample collection. All clinical cases were submitted for diagnosis and post-mortem examination at the Kasetsart Veterinary Diagnostic Center, Faculty of Veterinary Medicine, Kasetsart University, Kamphaeng Saen Campus, Nakhon Pathom, between 2021 and 2023. All *R. anatipestifer* isolates were cultured on tryptic soy agar (Difco Laboratories, USA) supplemented with 5% sheep blood and incubated at 37°C for 24 h under microaerophilic conditions (5% CO_2_). Suspected colonies were subjected to Gram staining and DNA extraction. The confirmed pure colonies were collected and stored in Cryobank (Mast Group, United Kingdom) at −80°C until use for further experiments.

### Molecular identification of *R. anatipestifer*

Isolate identity was confirmed by polymerase chain reaction (PCR) amplification and sequencing of the *16S ribosomal RNA* gene using species-specific primers [9]. Positive control of *R. anatipestifer*, which was confirmed through DNA sequencing and preserved as our positive strain, was included in all PCR tests.

### Determination of minimum inhibitory concentrations (MICs) of quinolones

The MICs for nalidixic acid, ciprofloxacin, and enrofloxacin (Sigma-Aldrich, USA) were determined for the 37 *R. anatipestifer* isolates using the standard agar dilution method, following the guidelines of the Clinical and Laboratory Standards Institute [10]. The MIC for each *R. anatipestifer* isolate against each of the three tested quinolone antibiotics was determined in triplicate. The antimicrobial agents were tested at concentrations ranging from 0.03 μg/mL to 128 μg/mL, except for nalidixic acid, which was tested between 0.12 μg/mL and 128 μg/mL. The quality control strains included *Escherichia coli* American Type Culture Collection (ATCC)® 25922 and *Pseudomonas aeruginosa* ATCC® 27853.

### DNA extraction

Genomic DNA was extracted using the boiling lysis method: [11] Colonies were suspended in 60 μL DNase/RNase-free water, heated at 100°C for 10 min, and centrifuged at 10,000× *g* for 10 min, and the supernatant was transferred to a new tube and stored at −20°C for further analysis. The primer sequences and amplicon sizes are presented in Table 1 [7, 9, 12, 13].

**Table 1:** Primers used in this study.

Target gene	Primer	Oligonucleotide sequence (5’- 3’)	Product size (bp)	References
*Riemerella anatipestifer* conformation				
*16s rRNA*	RA-F RA-R	CAGCTTAACTGTAGAACTGC TCGAGATTTGCATCACTTCG	665	[9]
Quinolone resistance genes				
*gryA*	gryA-F gryA-R	AGAGAAGGGTTTTGTATGG GGGGGCTTCAGTATAACGCA	241	[7]
*parC*	parC-F parC-R	GACCGTGCCATTCCTTCTA AGCCCTACACCAATACCTTCC	416	[12]
*qnrA*	qnrA-F qnrA-R	ATTTCTCACGCCAGGATTTG GATCGGCAAAGGTTAGGTCA	516	[13]
*qnrB*	qnrB-F qnrB-R	GATCGTGAAAGCCAGAAAGG ACGATGCCTGGTAGTTGTCC	469	[13]
*qnrS*	qnrS-F qnrS-R	ACGACATTCGTCAACTGCAA TAAATTGGCACCCTGTAGGC	417	[13]

*16s rRNA*=*16S ribosomal RNA*

### PCR amplification of gyrA and parC genes

PCR reactions (20 μL) contained 10 μL of 2× DreamTaq Green Master Mix (Thermo Fisher Scientific, USA), 0.25 μM of each primer, 7 μL of nuclease-free water, and 10 ng of DNA template. The same thermal cycling conditions were used for both targets, except for the annealing temperatures, which were 52°C for *gyrA* and 56°C for *parC*. The protocol consisted of initial denaturation at 96°C for 5 min, followed by 30 cycles of denaturation at 94°C for 1 min, annealing at 52°C for *gyrA* and 56°C for *parC* for 50 s, and extension at 72°C for 1 min. A final extension was performed at 72°C for 10 min. PCR amplicons were visualized by electrophoresis on 1.5% agarose gels (Vivantis, Malaysia) in 1× Tris-borate-ethylenediaminetetraacetic acid buffer (Thermo Fisher Scientific) and stained with OnePCR Ultra (GeneDireX, Inc., Taiwan). Positive PCR products were purified using MEGA quick spin plus (iNtRON Biotechnology, South Korea) and confirmed by DNA sequencing using the Sanger technique (Bionics, Republic of Korea).

### Detection of *PMQR* genes

The presence of *PMQR* genes (*qnrA*, *qnrB*, and *qnrS*) was assessed using PCR, as described by Robicsek *et al*. [14]. The amplification process consisted of an initial denaturation step at 94°C for 5 min, followed by 30 cycles of denaturation at 94°C for 45 s, annealing at 54°C for 45 s, and extension at 72°C for 1 min. The PCR reactions included a final step at 72°C for 5 min to complete product elongation. PCR products were analyzed using the method previously described by Robicsek *et al*. [13].

### Sequence analysis and mutation detection

Sanger sequencing results were analyzed using the basic local alignment search tool (https://blast.ncbi.nlm.nih.gov/Blast.cgi) to confirm the identity of the target sequences. All PCR products were sequenced and compared with the wild-type *R. anatipestifer* ATCC 11845 *gyrA* and *parC* gene sequences (accession number NC_014738) [15]. Mutation and phylogenetic cluster analysis were performed using BioEdit version 7.2.5 [16] and MEGA 11 [17].

## RESULTS

### MIC values for nalidixic acid, ciprofloxacin, and enrofloxacin

Table 2 shows the distributions of MICs for ciprofloxacin, enrofloxacin, and nalidixic acid, along with their MIC_50_ and MIC_90_ values. The MIC distribution for nalidixic acid ranged from 16 μg/mL to ≥128 μg/mL, with 16 isolates showing MIC values ≥128 μg/mL. The MIC_50_ and the MIC_90_ for nalidixic acid were 64 μg/mL and ≥128 μg/mL, respectively.

**Table 2:** MIC distribution for quinolones in *Riemerella anatipestifer* isolates (n = 37).

ABO^a^	Concentration (µg/mL)	Number of isolates with MIC (µg/mL)													MIC_50_ (µg/mL)	MIC_90_ (µg/mL)

		≤0.03	0.06	0.125	0.25	0.5	1	2	4	8	16	32	64	≥128		
NAL	0.12–128										3	9	9	16	64	≥128
CIP	0.03–128						1	21	9	6					2	8
ENR	0.03–128				1		1	2	33						4	4

a NAL=Nalidixic acid, CIP=Ciprofloxacin, ENR=Enrofloxacin, MIC=Minimum inhibitory concentration, ABO: Antibiotic

For ciprofloxacin, MIC values ranged from 1 μg/mL to 8 μg/mL, with 21 isolates exhibiting an MIC of 2 μg/mL. The MIC_50_ and MIC_90_ values for ciprofloxacin were 2 μg/mL and 8 μg/mL, respectively.

Enrofloxacin MICs ranged from 0.25 μg/mL to 4 μg/mL, with 33 isolates having an MIC of 4 μg/mL. The MIC_50_ and MIC_90_ values for enrofloxacin were 4 μg/mL.

### Detection of *PMQR* genes and QRDR mutations in GyrA and ParC

**Figure 1:**
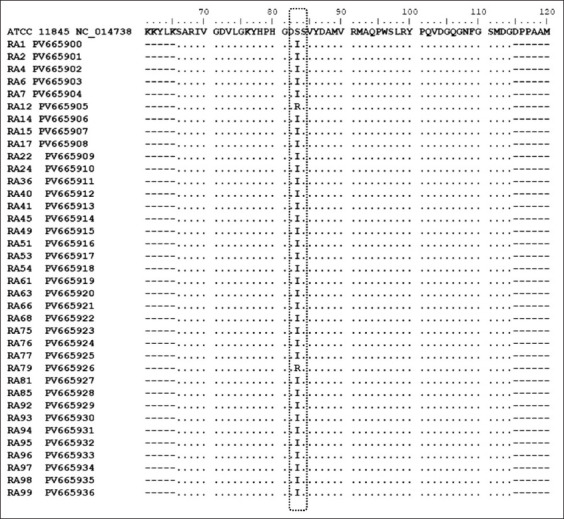
Amino acid alignment for the QRDR of GyrA of *Riemerella anatipestifer* isolates from ducks (37 isolates). The black box indicated amino acid substitution at codon 83 of the QRDR in GyrA compared to the wild-type *R. anatipestifer* ATCC 11845. QRDR=Quinolone resistance-determining region, ATCC=American Type Culture Collection.

None of the *R. anatipestifer* isolates harbored *qnrA*, *qnrB*, or *qnrS* genes analyzed in this study. However, all isolates exhibited a single mutation in *gyrA* at codon 83, with 35 harboring the C248T mutation and two harboring the C248G mutation. These mutations resulted in amino acid changes at codon 83, producing either a Ser83Ile or Ser83Arg substitution (Figure 1).

No mutations were identified in the *parC* gene. The observed MIC values for ciprofloxacin ranged from 1 μg/mL to 8 μg/mL, for enrofloxacin from 0.25 μg/mL to 4 μg/mL, and nalidixic acid from 16 μg/mL to 128 μg/mL. A summary of these findings is provided in Table 3.

**Table 3:** Quinolone MICs and associated *gyrA* and *parC* mutations in *Riemerella anatipestifer* isolates (n = 37).

Number of isolates	QRDR mutation^a^		MIC of quinolones (number of isolate)		
	
	*gyrA*	*parC*	NAL^b^	CIP	ENR
35	C248T (Ser83Ile)	-	16 (2), 32 (9), 64 (9), 128 (15)	1 (1), 2 (19), 4 (9), 8 (6)	0.25 (1), 1 (1), 2 (1), 4 (32)
2	C248G (Ser83Arg)	-	16 (1), 128 (1)	2 (2)	2 (1), 4 (1)

a Nucleotide change in *gyrA* (amino acid substitution in *GyrA*),

b NAL=Nalidixic acid, CIP=Ciprofloxacin, ENR=Enrofloxacin, QRDR=Quinolone resistance-determining region, MIC=Minimum inhibitory concentration

### Phylogenetic analysis of the *gyrA* gene

**Figure 2:**
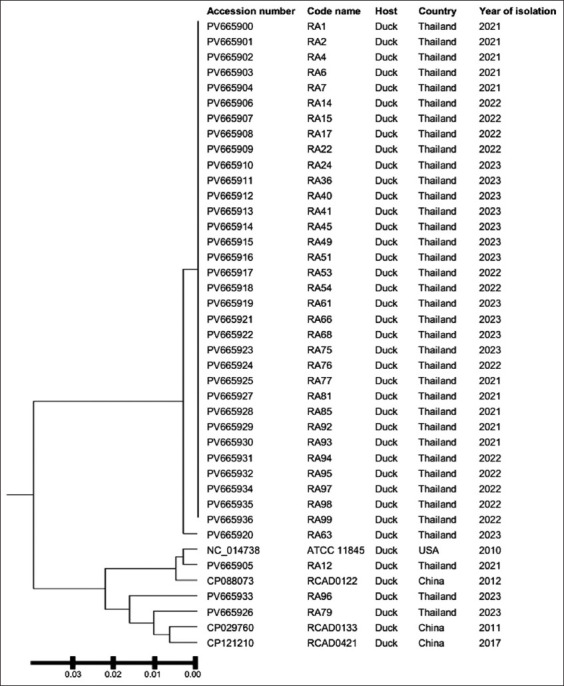
Phylogenetic analysis constructed by the unweighted pair group method using arithmetic averages method using the nucleotide sequences of the QRDR in *gyrA*. The isolates from this study are compared with four reference strains of *Riemerella* *anatipestifer*, including *R. anatipestifer* ATCC 11845, *R. anatipestifer* CP088073, *R. anatipestifer* CP029760, and *R. anatipestifer* CP121210. QRDR=Quinolone resistance-determining region, ATCC=American Type Culture Collection.

In addition, this study conducted a phylogenetic analysis of the *gyrA* gene in comparison to GenBank database sequences. This result revealed four primary clusters (Figure 2).

Thirty-four isolates from Thailand were mainly grouped in Cluster I. Only one isolate (RA63) was detected in Cluster II. The isolate RA12 was grouped in Cluster III, along with the wild-type strain (ATCC 11845) and the isolate obtained from China. Cluster IV included the remaining two isolates (RA79, RA96), which were in close proximity to the isolates from China.

## DISCUSSION

This study aimed to investigate the molecular mechanisms of quinolone resistance in *R. anatipestifer* isolates from ducks in Thailand by analyzing MIC profiles, mutations in QRDR regions of *gyrA* and *parC*, and the presence of *PMQR* genes.

The emergence of quinolone resistance in Gram-negative bacteria such as *E. coli* and *Salmonella* is well-documented and poses a significant threat to poultry health. *R. anatipestifer*, a bacterial pathogen primarily affecting ducks, is responsible for fibrinopurulent polyserositis, characterized by lesions in multiple visceral organs. However, the mechanisms underlying quinolone resistance in *R. anatipestifer* remain poorly understood.

### QRDR mutations and MIC trends

In this study, all *R. anatipestifer* isolates exhibited a QRDR mutation in the *gyrA* gene, specifically at codon 83, whereas no mutations were identified at codon 87 of *gyrA* or within the *parC* gene. MIC values for nalidixic acid ranged from 16 μg/mL to 128 μg/mL, whereas MIC values for ciprofloxacin and enrofloxacin varied from 0.03 μg/mL to 8 μg/mL and 0.03 μg/mL to 4 μg/mL, respectively.

The findings are consistent with a previous study by Sun *et al*. [7], which identified Ser83 and Asp87 in GyrA as mutation hotspots in *R. anatipestifer*, correlating with increased MICs for nalidixic acid, ciprofloxacin, and enrofloxacin compared with wild-type strains. The same study also suggested that double mutations may result in higher MICs than single mutations. Furthermore, phylogenetic analysis of *gyrA* demonstrated high homology among our isolates, indicating the widespread circulation of these mutated strains within our study area.

### Cross-species similarities in resistance mechanisms

Similar to patterns observed in other Gram-negative bacteria, our study confirmed that a single mutation at codon 83 (Ser83Ile and Ser83Arg) in GyrA follows a pattern similar to that observed in other Gram-negative pathogens. This finding is supported by research on *Salmonella* conducted by Koide *et al*. [18], who showed that the Ser83Ile mutation in GyrA reduced quinolone-binding affinity and contributed to high-level quinolone resistance. Furthermore, the substitution of serine with leucine at codon 83 has been frequently reported in quinolone-resistant *E. coli* strains [19, 20].

### Absence of *parC* mutations and literature comparisons

The absence of *parC* mutations in this study contrasts with previous reports by Sun *et al*. [7] and Zhu *et al*. [21]. Sun *et al*. [7] reported that high ciprofloxacin MICs (MIC ranging from 16 mg/mL to 64 mg/mL) may be linked to an amino substitution at Arg120Glu in ParC, as isolates with mutations in both *gyrA* and *parC* exhibited higher ciprofloxacin MICs than those with *gyrA* mutations alone. However, in our *R. anatipestifer* isolates, ciprofloxacin MICs remained below 8 μg/mL. In addition, Zhu *et al*. [21] identified high-frequency variations in ParC, specifically Val799Ala and Ile811Val, although these modifications did not significantly impact fluoroquinolone resistance.

### Regional context and QRDR evolution

Research on QRDR mutations in *R. anatipestifer* has not been conducted in Thailand; however, studies on QRDRs in Gram-negative bacteria have been reported. Sinwat *et al*. [22] identified amino acid alterations at codons 83 and 87 (Ser83Tyr, Asp87Tyr) in quinolone-resistant *Salmonella enterica* isolates from chicken meat, pork, and humans. Mutations in *gyrA* (codons 83 and 87) and *parC* have been detected in avian pathogenic *E. coli* isolates from broilers and native chickens [23]. These findings suggest that selective pressure in the region facilitates the adaptation and evolution of QRDR mutations in *R. anatipestifer*, a phenomenon similar to that observed in other bacteria. Additional studies employing site-directed mutagenesis of QRDR in *R. anatipestifer* are necessary to identify the precise mutation sites and their role in quinolone resistance.

### Absence of *PMQR* genes and comparative insights

Members of the *QNR* gene family are widely reported in Gram-negative bacteria in poultry [24, 25]. This study investigated the presence of *qnr* genes; however, no *R. anatipestifer* isolates carried *qnrA*, *qnrB*, or *qnrS*. Consistent with our findings, Lyu *et al*. [26] reported the absence of the *qnrS* and *aac(6’)-Ib-cr* genes in *R. anatipestifer* isolates from Shandong Province, China. Moreover, another study in China found that 103 *R. anatipestifer* isolates were negative for the *qnrA*, *qnrB*, *qnrC*, *qnrD*, *qnrS*, *qepA*, and *oqxAB* genes [7].

Considering the scarcity of PMQR data on *R. anatipestifer* and the small sample size in this study, further investigations are warranted to identify novel or underreported quinolone resistance genes using previously recognized *PMQR* genes in *R. anatipestifer*, such as *aac(6’)-Ib-cr* (encoding quinolone-modifying enzymes), *oqxAB* (encoding efflux pumps), or other *qnr* variants [27]. Advanced molecular techniques, such as next-generation sequencing, are needed for their identification.

### Public health significance and policy implications

Because of the importance of quinolones to human and animal health, monitoring resistance in *R. anatipestifer* in ducks is crucial. Poultry production significantly contributes to the Thai economy, and the Thai government recognizes the importance of addressing antimicrobial resistance (AMR), imple-menting regulations that control the use of quinolones in livestock.

Thus, the results of this study provide preliminary data on quinolone susceptibility and some resistance mechanisms in *R. anatipestifer*. This information can serve as the basis for developing and updating the national AMR plan regarding quinolone use in poultry. At present, there are no standardized clinical breakpoints for quinolones against *R. anatipestifer*. The continuous surveillance of quinolone resistance with a larger number of isolates from different regions in Thailand is necessary. This will help establish region-specific breakpoints, and trends in MIC values can guide decisions about the use of quinolone treatment for *R. anatipestifer* infections in duck farms.

## CONCLUSION

This study provides the first molecular insight into quinolone resistance mechanisms in *R. anatipestifer* isolates from ducks in Thailand. All 37 isolates exhibited mutations at codon 83 of the *gyrA* gene, either C248T (Ser83Ile) or C248G (Ser83Arg), while no mutations were detected in *parC*, and none of the isolates carried *PMQR* genes, such as *qnrA*, *qnrB*, or *qnrS*. The MICs ranged from 16 μg/mL to ≥128 μg/mL for nalidixic acid, 1–8 μg/mL for ciprofloxacin, and 0.25–4 μg/mL for enrofloxacin, with elevated resistance levels clearly associated with *gyrA* mutations.

These findings have practical implications for improving antimicrobial stewardship in poultry health management. The identification of codon 83 in *gyrA* as a resistance determinant offers a molecular marker for rapid diagnostic screening and targeted treatment decisions in duck farms. Moreover, the absence of *PMQR* genes in these isolates suggests that resistance in *R. anatipestifer* is currently dominated by chromosomal mutations, which simplifies molecular tracking and surveillance efforts.

This study is strengthened by its integration of phenotypic MIC data with genotypic characterization and phylogenetic analysis, providing a comprehensive perspective on resistance evolution. It also benefits from the use of validated molecular tools and standardized susceptibility testing protocols. However, limitations include a relatively small sample size that may not reflect nationwide trends, and the restricted *PMQR* gene panel, which excluded other important resistance determinants such as *aac(6’)-Ib-cr* or *oqxAB*. In addition, the lack of established clinical breakpoints for *R. anatipestifer* hinders the definitive classification of resistance levels.

Future research should involve larger, regionally diverse isolate collections and employ next-generation sequencing to identify novel resistance genes or mechanisms. Functional validation of QRDR mutations through site-directed mutagenesis is also warranted to confirm their role in resistance.

In conclusion, this study demonstrates that quinolone resistance in *R. anatipestifer* in Thailand is primarily associated with a conserved mutation at codon 83 of the *gyrA* gene. These findings provide essential baseline data to support rational quinolone use, guide surveillance strategies, and inform the development of national AMR policies targeting duck production systems.

## AUTHORS’ CONTRIBUTIONS

CP: Performed the experiments, managed the data collection and analysis, and drafted and reviewed the manuscript. SL, KW, SK, PP, and TS: Collected the samples and performed the experiments. NS: Conceptualized and designed the study, performed experiments, and revised the manuscript. All authors have read and approved the final manuscript.
